# Design, Implementation, and Adaptation of a Tutoring Program for the Competency Development of New Nurses in a Hospital Emergency Department

**DOI:** 10.3390/nursrep14030176

**Published:** 2024-09-14

**Authors:** Marta Manero-Solanas, Noelia Navamuel-Castillo, Silvia Garcés-Horna, Nieves López-Ibort, Carmen Angustias Gómez-Baca, Ana Gascón-Catalán

**Affiliations:** 1 Hospital Universitario Miguel Servet, 50009 Zaragoza, Spain; nnavamuel@salud.aragon.es (N.N.-C.); sgarcesh@salud.aragon.es (S.G.-H.); 2 Instituto de Investigación Sanitaria de Aragón, 50009 Zaragoza, Spain; nlopezi@salud.aragon.es (N.L.-I.); cagomez@salud.aragon.es (C.A.G.-B.); 3 Hospital Clínico Universitario Lozano Blesa, 50009 Zaragoza, Spain; 4 Departamento de Fisiatría y Enfermería, Facultad de Ciencias de la Salud, Universidad de Zaragoza, 50009 Zaragoza, Spain

**Keywords:** methodological approach, mentoring, clinical nurse, competence, professional development, self-assessment, evaluation

## Abstract

The healthcare environment faced by nurses is complex, with high workloads and situations of high comorbidity. The integration of nurses into the work environment is a cause for concern, and improvements are sought for their incorporation into the workforce. The benefits of mentoring programs are described not only for nurses but also for patients with safer practices and benefits for the institution due to the increased commitment of its professionals. A methodological article that illustrates the complete process to design and implement a tutoring program for new nurses in an emergency department is presented. The competency profile required for the figure of tutor was developed, including the steps followed for the development of the program and the structure and phases of which it is composed, as well as the validation of the evaluation instruments of the process. A strength was the participation of experts during the in-depth analysis of the competency profile, as well as in the adaptation of the evaluation items, which endorses the pertinence, relevance, usefulness, and clarity of the content of this program. The transparency in this methodology makes it possible to follow the steps for its reproduction and applicability in other hospitals.

## 1. Introduction

### 1.1. Problem Description

The quality of care and patient safety have become key points of current health policies, where the user is placed at the center of the system. This quality of care depends to a large extent on the skills that its professionals develop. In Spain, universities [1] are the first to guarantee the minimum acquisition of skills for their graduates; however, in order to be able to talk about a safe and effective practice in the workplace, it is necessary to ensure, from the hospitals themselves, that professionals acquire and develop the necessary skills for their jobs. The transition from the university to the professional role is documented in the history of nursing, within its theoretical framework, by authors such as Benner (1984) with his theory from novice to expert. 

According to Benner [2], there are five professional levels or degrees of competence acquisition: beginner/novice, where the nurse faces new situations with no prior experience; advanced beginner, where the nurse starts to recognize and study clinical situations with greater skill; competent, where the nurse can imitate others, recognize patterns, prioritize care, and develop standardized plans; proficient, where the nurse has an intuitive understanding of situations and becomes more involved with the patient and their family; and expert, where the nurse intuitively masters care, quickly identifies problems, and adapts care plans to meet patient needs. In the clinical world, beginners and advanced beginners often struggle with task analysis, managing concurrent demands, and prioritizing clinical situations and interventions. As a consequence, they delegate the difficult situations they face to more experienced clinical colleagues, trusting that they automatically know how to solve those problems [3]. Benner’s model continues to be an international benchmark [4,5] and consequently a theoretical framework of reference for our study, focused on nurses who join the hospital emergency department. 

### 1.2. Available Knowledge

Authors such as Myers, 2010 [6] and Arrowsmith, 2016 [7] reflect in their studies on the incorporation of novice nurses into the labor market, uncertainty about the technical aspects of nursing care, the lack of critical thinking, and the inability to think holistically, which translates into situations of insecurity and stress. These perceptions have been described in different studies [8,9] on nurses’ adaptation to the work environment. Authors such as Marrero [10] and Ten Hoeve [11] have given voice to nurses, showing that it is a totally topical problem, in which they describe problems in adapting to the professional role and situations of fear and insecurity when they feel alone in the task to be carried out and include in their proposals to improve this situation tutoring programs in the work environment.

Since the 1970s, techniques and strategies for recruiting and training employees have been developed in private companies, such as mentoring, coaching, empowerment, and counseling [12,13,14]. In this project we have considered it more appropriate to use the term “tutoring” because it is closer, more didactic, and more in line with the public health system. 

The general objective of the tutoring is to guide new professionals, ensuring basic training of the service in which they are going to work, which includes the user profile and their main health demands, the protocols of the unit, the technical skills required, and the most common care tasks. However, a mentoring program includes a broader concept in which new staff acquire proficiencies and become part of a work group, receiving support and advice from their mentor [15,16].

Integration into the work environment is a long-standing concern within the nursing profession as described by El Haddad [17], and although it is not a new discourse, it is no less important. In fact, and perhaps because the healthcare environment faced by nurses is complex, with high workloads [18] and situations of high comorbidity [19], the discourse seems to be increasingly audible. There are many authors [20,21,22,23] who propose improvements for labor incorporation and point out the importance of promoting the figure of the clinical tutor, to offer opportunities and resources to new staff and allow them to grow professionally, gaining autonomy and security in the development of clinical skills.

The benefits of tutoring programs have been described not only for nurses, in which their competencies, confidence, and satisfaction improve, but also for the patient [24], with safer practices, and for the institution [25] due to the increase in the commitment of its professionals and the decrease in costs due to a better use of resources. The benefits are, in addition, identified as evidence of structural empowerment [26]. 

### 1.3. Rationale

Hospital emergency services (EDs), an area of interest for our study, as a gateway to the hospital environment, are units designed to provide highly professionalized medical treatment, with immediate availability of resources, to patients who require urgent care, at any time of the day or night [27].The ED directly interacts with the central services of the hospital to carry out complementary examinations, mainly laboratory and radiodiagnosis, and with the rest of the clinical care and specialized medical services, if required.

The defining characteristics of emergency services, in addition to showing the high care burden of the nursing staff, indicate the complexity of the role of the emergency nurse, in situations of great clinical variability and high specialization.

The increasing complexity of healthcare organizations necessitates finding cost-efficient and effective responses to new societal challenges. New nursing profiles and roles should be studied and developed that are capable of adapting to these new needs and ensuring the provision of high-quality care in safe environments [28]. 

Based on all of the above, it is surprising that there is no regulated figure of the clinical tutor for nursing in Spain, such as that widely developed in Europe, whose origins are linked to the British public system, where the figure of the preceptor was born in the 1960s [15]. The development and evaluation of mentoring programs for nursing staff in areas of critical patient care such as hospital emergency services are essential.

### 1.4. Aim

The present study aimed to detail the development and validation process of a tutoring program for new nurses in an emergency department of a referral hospital, with an emphasis on the expert validation. This study contributes to the existing body of knowledge by providing a validated, expert-reviewed tool for objectively assessing the competency development of novice nurses in an emergency department, which can be integrated into similar tutorship programs to enhance training effectiveness in clinical settings.

## 2. Materials and Methods

This paper presents a complete process to design and implement a tutoring program for new nurses in an emergency department. For the development of this manuscript, the structure and guidelines provided by SQUIRE (Standards for Quality Improvement Reporting Excellence) were followed. The competency profile required for the figure of tutor is described, including the steps followed for the development of the program and the structure and phases of which it is composed, as well as the validation of the evaluation instruments of the process. Self-assessment tools are presented to contribute to the development of evaluative capacity and the acquisition of greater autonomy and awareness of the individual competency level, as well as the competency development rubrics, for objective evaluation by an external evaluator. The detailed description of the followed procedure allows its implementation in other emergency departments and could be used to incorporate the figure of tutor in other hospitals. 

### 2.1. Context

The ED where this procedure was implemented provides care to an average of 500 patients per day and is the reference center for around 400,000 inhabitants. It has an area of approximately 5000 m^2^, with a multidisciplinary team of professionals, and has a computer support system from which patient care is managed, known as PCH (Hospital Clinical Post).

The emergency unit is described in relation to three areas: the reception of the user, the assistance, and the observation and treatment of the patient. Access to the service is through the emergency admission station and then goes through a triage system, where the patient is assessed and classified, assigning priority through filtering protocols and a preliminary clinical assessment process.

Once classified, the patient is referred to a care area, determined by the priority and reason for consultation, and may be referred to the resuscitation area (immediate and emergency assistance, levels I and II); the traumatology area (musculoskeletal and joint pathology as a result of trauma); the boxes area (multiple medical pathologies); the outpatient consultation area for less urgent priorities; and the specialty area, with boxes for ophthalmology, otorhinolaryngology, urology, and surgery.

Once the patient has been assessed and cared for, they can be discharged home, admitted to the ward, or referred to an observation and treatment area, with a total capacity of 60 observation beds.

### 2.2. Interventions and Study of the Interventions

Once the unit and hospital where the procedure has been implemented have been contextualized, we describe the phases of the process that is structured in two parts, the profile of the tutor figure and the phases of the program designed and implemented with the tools created for the evaluation of skills and competencies of new nursing staff in an ED.

#### 2.2.1. Define the Competency Profile of the Figure of the Tutor

Nurse tutors exercise clinical leadership in the scope of their work, with autonomy to make complex decisions, based on the application of evidence and research results to their professional practice. In their practice, they integrate four roles: expert clinician, consultant, teacher, and researcher [29]. To access the position of tutor, expert knowledge in the area of care for which it is defined is required, as well as specific training, accredited and acquired through continuous training or specific postgraduate training, and clinical experience, a minimum of time in the clinical area considered [28], 5 years in our program, in such a way that the minimum competencies that the position outlined in the corresponding tutoring program should have can be ensured.

To define the competency profile of the tutor figure, a panel of experts in critical patient care was selected, made up of 10 nurses and supervisors who were experts in critical patient care. The inclusion criteria to be part of this panel of experts were defined by the hospital’s competency development area and included a minimum of 5 years of experience in critical patient care. The panel based its work on Martha Alles’ methodology and her dictionary of competencies [30]. Once consensus was reached, each competency was assigned a level, with 1 being the most basic level and 4 being the one considered as a reference. For the assignment and belonging to each level, observable behaviors were objectified. The profile was defined by four generic, four specific, and four technical competencies that are shown in Supplementary Material File S1.A, Table. 

Having reflected the methodology for defining the tutor’s profile, we now detail the tutoring program implemented.

#### 2.2.2. Reception and Tutoring Program Aimed at New Nurses in Hospital Emergency Department

The program consisted of two phases:

Phase 1: Welcome program: The welcome program was a reception program that aimed to welcome the person who had just joined and accompany them in the process of integration into the institution, with initial training in the basic elements.

Definition: This phase was defined as a set of activities carried out to provide new staff with the necessary information and knowledge about the organization they have become part of.

Objective: The objective was to facilitate the adaptation of the new professional and guarantee quality care, with the hosting from the tutor.

**Figure 1:**
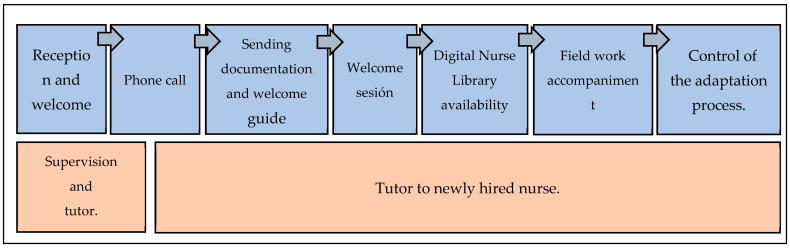
Reception and tutoring program. Phase 1: welcome program.

Structure: The content and structure of the program are presented in Figure 1. Further details can be found in Supplementary Material File S1.B of this manuscript, which also includes the individual program monitoring form (Supplementary Material File S1.C).

### 2.3. Measures

Completion of questionnaires: Before joining the service, questionnaires were sent to help the tutor to know in a more in-depth way what the real situation of the newly incorporated nurse was.

The objective was to evaluate what the starting point was and to create more effective lines of action based on the information obtained after the face-to-face interview [31,32]. The questionnaires to be completed were as follows:

Perceived stress: This questionnaire aimed to take a snapshot of the worker’s state of stress and anxiety at the current moment coinciding with their imminent incorporation. The instrument chosen, the Perceived Stress Scale (PSS) by Cohen, Kamarck, and Mermelstein (1983) [33], is one of the most widely used instruments for stress assessment. Its Spanish version, validated by Remor [34], was the one selected for our study. It consisted of 14 items with a 5-point Likert scale format: 0, “never”; 1, “almost never”; 2, “occasionally”; 3, “often”; and 4, “very often” [35]. The total PSS score was obtained by adding the total score of the 14 items. The obtained direct score indicated that a higher score corresponded to a higher level of perceived stress.

Perceived quality of professional life: This questionnaire addressed how the interested party felt in its direct relationship with their work situation. The questionnaire on perceived quality of professional life, designed and validated by Cabezas 1998 [36], was used. This questionnaire assessed the dimensions of workload, intrinsic motivation, and managerial support. It consisted of 35 questions, which were answered on a scale of 1 to 10, where 1 meant nothing and 10 meant a lot, and provided a summary measure of the perception of the quality of professional life. The application of this instrument can be individual or collective and has construct validity and reliability [37].

Question 35 of the professional quality questionnaire referred to the support of colleagues in the case of managerial responsibilities; the bibliography reflects [38,39] that in those studies in which the population under study did not hold this position, it was withdrawn or replaced. In our case, it was replaced by a question of pride and belonging: “I am proud to belong to this hospital”.

For the assessment of stress and perceived quality of professional life, the questionnaires were given upon the arrival at the emergency department of the new nurse, and at the year of contract or at the time of the end of their contract in the unit, to obtain information on the pre- and post-tutoring program. The self-assessment tool agreed upon by the panel of experts is detailed in the results section and more specifically in the Supplementary Material File S1.D, Tool.

Phase 2: A Competence Development Program: This phase aimed to provide on-the-job training to the new recruit in order to develop the skills and competencies necessary for the performance of the tasks of the same, supported by the support and advice of the figure of a tutor.

Definition: This phase was defined as a set of processes for the competency assessment of emergency nurses and training activities designed to reduce the learning curve and the development of nurses’ competencies in certain skills and competencies of the nurse of critically ill patients.

Objective: The objective was to make it easier for each professional to develop the competencies defined for their position, training them through a free and voluntary training program, with the unit itself being the one that self-manages the development of these competencies to each professional group with a criterion of qualification and cost-effectiveness.

Structure: It was a training intervention, and participation was completely voluntary. An initial baseline test of technical competencies was carried out on all newly incorporated nurses. After this initial assessment, five training workshops were offered for the development of nurses in these competencies, and the same test was repeated every month, to assess whether there had been an improvement compared with the baseline test in these competencies. The same exercise were repeated after 3 months, for the third and last time, to check if the acquisition of skills was maintained over time and the knowledge had been internalized.

### 2.4. Analysis

For the objective evaluation of competency development, a tool was developed that allowed us to objectively evaluate the acquisition of competencies and technical skills of new nurses by an external evaluator. Evaluation rubrics and diagrams were created, which were assessed and approved by a panel of experts. This panel of experts consisted of 20 nurses who were experts in the care of critical patients in the emergency room, who were selected based on accessibility and experience criteria, at least 5 years as a nurse care in the ED. In-person meetings were held to present the program. The final version of the program was reached by consensus. The content validity index was evaluated through an online questionnaire completed by the panel of experts.

For the validation of competency assessment rubrics, an ad hoc form was created, similar to other competency assessment validation instruments in the framework of teacher professional development [40], which presented questions about whether the rubrics described the desired level for each level, whether the progression between the different competency levels could be objectified, whether they were observable and identifiable principles for the assessor, whether the competency levels were consistent with the progress in the care development of the ED nurse, and whether they believed or wanted to add or remove any descriptors, as well as allowing respondents to provide any comments or observations in an open text.

We detail the competency assessment tool in our results, so that they can be replicated in new scenarios.

### 2.5. Ethical Considerations

The study was conducted in accordance with the Declaration of Helsinki and approved by the Clinical Research Committee of Aragon (PI 23/2013) for studies involving humans.

## 3. Results

A first objective of the work was to describe the competence profile of the tutor. The selection of competencies, made by the panel of experts, reflected that the tutor must have the generic competencies of commitment (level 4), quality and innovation (level 3), ethics (level 3), and user and professional guidance (level 3); the specific competencies of analytical thinking (level 3), teamwork (level 3), effective communication (level 3), and continuous learning (level 3); and the technical competencies of coach (level 4), collaboration (level 2), empowerment (level 3), and temper and dynamism (level 3) (Supplementary Material File S1.A, Table).

Having described the competency profile of the tutor, we show in our results the questionnaire created for the self-assessment of the competency profile of the emergency nurse.

The self-assessment questionnaire was based on the competencies selected by agreement of the panel of experts, to define the competency profile of the emergency nurse. The selected competencies were initiative/autonomy, effective communication, tolerance to pressure, and teamwork. Their definitions as part of the assessment tool are described below:

- Initiative Autonomy: Initiative autonomy is the ability to act proactively and devise and implement solutions to new problems and/or challenges, with decisiveness and independence of judgment. It implies the ability to respond quickly, effectively, and efficiently to new requirements. All this is achieved by making the most of the resources and opportunities that arise in the environment.
- Effective Communication: Effective communication is the ability to actively listen and clearly transmit to users, family members, and professionals the appropriate information for their needs and demands and to maintain open communication channels that guarantee their rights and duties dictated by the organization.
- Pressure Tolerance: Pressure tolerance is the ability to respond and work effectively under adverse conditions, weather, or overload, in situations of high demand and pressure. It involves the ability to relieve stress in a way that is acceptable to the individual, other people, and the organization. It is the ability to control personal emotions and avoid negative reactions to provocations, opposition, or hostility from others or when working under stressful conditions.
- Teamwork: Teamwork is the ability to collaborate with others, be part of a group, and work with other areas of the organization in order to achieve, together, the organizational strategy, subordinating personal interests to group objectives. It involves having positive expectations of others, understanding others, and creating and maintaining a good work environment.

The competencies defined for the emergency nurse were the basis for the assessment of novice staff upon their arrival at the emergency department and their entry into the tutoring program.

A questionnaire was designed to collect the four defined competencies and the 21 nursing skills associated with the competency profile of the emergency nurse, using the dictionary of competencies by Martha Alles [30]. Each skill and competency consisted of five answer options, Benner’s five levels [2,3] of experience in clinical practice, from beginner to expert, and whose concepts were included in the self-completion questionnaire (see in Supplementary Material File S1.D, Tool). To define the competency profile of the emergency nurse, the meetings were in person, once a week, and consensus was reached after 10 meetings. The competency profile reached by consensus was subsequently validated with a questionnaire sent to the experts by email, reaching a CVI (content validity index) value = 0.877.

### Technical Competencies and Skills Assessment Rubrics

These rubrics were applied in a mock test designed for competency assessment. Each rubric had a description of the evaluation criterion observable at each level based on the methodology already referenced in our work by Benner and also had its own application diagram that facilitated and homogenized the evaluation. The competencies proposed by the panel of experts for critically ill patients were selected and were chosen for their high specificity in the service and their time-dependent relationship in terms of their execution since their application usually occurs in emergency contexts. The created competency assessment rubrics were in accordance with the definition of the minimum contents in the training of emergency nurses, published and available in the consensus document of the Spanish Institute for Nursing Research and the General Council of Nursing of Spain, together with the Spanish Society of Medicine and Nursing for Emergencies and Emergencies SEMES [41].

To validate the rubrics, they were presented to a panel of experts in meetings that were in person. Consensus was reached after two meetings. The final version of the rubrics was subsequently validated with a questionnaire sent to the experts by email, reaching a CVI (content validity index) value = 0.9. At the time when the rubrics were analyzed, evaluated, and validated by the panel of experts regarding their adequacy, the contributions of the panel were reviewed, to include those descriptors that were provided, reformulating some levels, to arrive at a definitive version and principles of applicability.

The competency assessment with this tool was based on the following principles:

- All rubrics will be applied with reference to the test for which they have been designed and to the activity that the assessed staff develops in front of the assessor in terms of practical skills.
- Competency level assignment diagrams have been developed to help in the application of the evaluation rubric for each technique. In its implementation, it is clarified that the competency level of competence is acquired when one has the theoretical knowledge and is able to imitate a pattern in the simulated test. One is considered to reach the level of efficiency when, in addition, one presents competence and adds an intuitive and general vision of the test one faces, anticipating complications and showing skill in the competencies and skills to be evaluated. And finally, to acquire an expert level, one must have experienced a real situation in a work environment, and have the ability to train other colleagues.
- One must go through all the scenarios that make up the test, keeping the numerical order reflected in each section of the evaluation diagram.
- If all the requirements necessary to assign a competency level accurately are not met, the immediately preceding level will be assigned.

**Figure 2:**
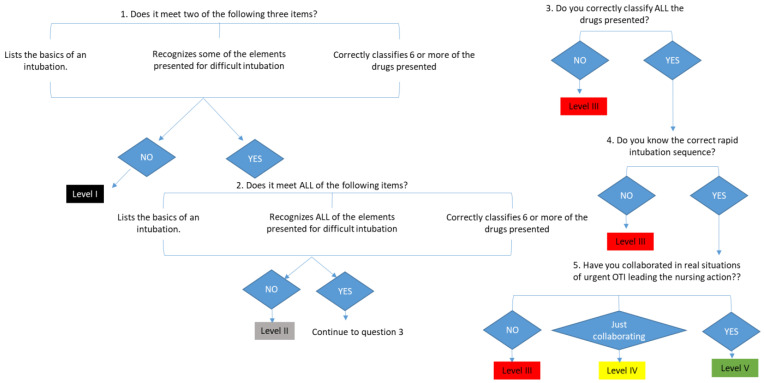
Flowchart for assessing the level of competence in the knowledge and management of invasive mechanical ventilation in emergency situations application diagram.

**Figure 3:**
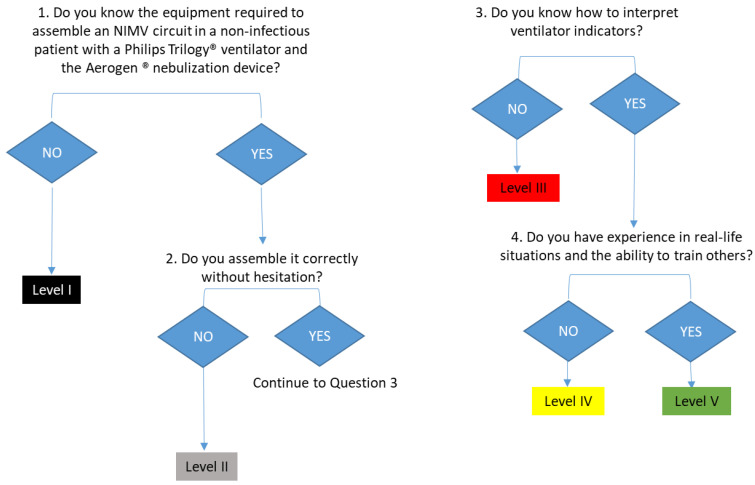
Flowchart for assessing the level of competence in the knowledge and management of invasive mechanical ventilation in emergency situations of evaluation of the level of competence in the knowledge and management of non-invasive mechanical ventilation (NIMV) and Aerogen^®^ vibrating mesh nebulization system application diagram.

Below are two of the competency assessment rubrics developed for the assessment of competence and airway management (Table 1 and Table 2). In order to facilitate its applicability, in addition to the rubric itself and its application flowchart (Figure 2 and Figure 3), the principles assigned and followed in each rubric individually are detailed and described, for their correct reproduction in a simulated environment. Competency assessment tools are under intellectual protection, and their authors should be contacted for use and reproduction.

1. Evaluation of the level of competence in the knowledge and management of invasive mechanical ventilation in emergency situations (Table 1 and Figure 2):

This test evaluates the knowledge and management of the orotracheal intubation technique in emergency situations. The nurse must identify the material needed for an orotracheal intubation. It is considered that the basic elements necessary to perform a basic orotracheal intubation are as follows: laryngoscope, orotracheal tube, guide or fastener, water-soluble lubricant, 10 mL syringe, stethoscope, resuscitator balloon with mask, gauze bandage, or other tube restraint device. In order for the section to be considered correct in its entirety, these 10 elements must appear in their entirety. Regarding the assessment of the knowledge of the material for the approach of the difficult airway, the nurse is presented with photographs of the material used in the approach of a difficult airway in orotracheal intubation. When viewing the photograph, the nurse must specify its name. In our case, we chose photographs of material available in our unit that corresponded to the following devices: Airtraq^®^, simple laryngeal mask, Fastrach, Frova, and ringed endotracheal tube. In order for this section to be valid, it is necessary that one specifies the name of the device and not just how the device is used or what the device is for. If necessary in a critical situation, it is important that the entire care team knows what the material is called in a specific way.

To assess the degree of knowledge about the management of drugs in the usual sequence of orotracheal intubation, the nurse has to classify the drugs according to the stage of use within the usual sequence of intubation. Ten drugs present in the unit are shown, and the nurse has to place at which stage of the intubation process each one is used, indicating whether it is used in premedication, sedation, or the relaxation phase of the patient.

2. Assessment of the level of competence in the knowledge and management of non-invasive mechanical ventilation (NIMV) (Table 2 and Figure 3):

This test evaluates the patient’s knowledge and management with NIV and the Aerogen^®^ vibrating mesh nebulization system.

To assess the knowledge of the material needed to assemble a NIV circuit, a case study is presented to the nurse. We detail whether it is a potentially infectious patient or not and the ventilator we would have. The nurse must deduce how to choose the right material and assemble it in the right way within the context described. In our case, we used the assumption of a non-infectious patient who was going to undergo NIV therapy with a Philips Trilogy ventilator^®^. The nurse must choose from the material available in the service. We consider the test to be the right one if the nurse chooses the right material and, in addition, this choice is argued in a reasoned way (interface, face shields, tubing, and filters). The assembly of the circuit will be considered valid if the nurse is able to assemble the circuit based on the reasoning used for the choice of the material in a correct way, under the given premises of the context and without hesitation.

To assess knowledge in the handling of the proposed nebulization system, the nurse must know the parts necessary for its assembly (a T-connection for tubing, an Aerogen^®^ system that contains the mesh and where the drug is inserted, an adapter cable for the controller with a USB output, and a connector to electricity). To evaluate the mounting of the device, the nurse will be provided with a properly mounted NIV circuit to which the nurse will need to attach the Aerogen^®^ device. To consider it correct, the nurse needs to attach it properly and without hesitation. The nurse also needs to explain how to load the necessary medication into it and put it into operation correctly.

In order to assume that it is correct that it is capable of interpreting indicators of the ventilator monitor and solving the incidents shown, we suggest that the nurse be shown a photograph of a ventilator in which there is an alarm, which they should interpret and indicate how it could be solved and what aspects should be reviewed as a result of its activation. In our case, we based ourselves on the reading and interpretation of a photograph in which an alarm appeared that suggested the detection of medium-high volumes within a context of NIMV in pressure control mode and asking them, from some proposed alternatives, to choose which of them should be reviewed when this type of alarm is detected. To pass this section, the nurse must indicate all the correct alternatives and none of the incorrect ones.

## 4. Discussion

The present study shows a detailed process for the implementation of a tutoring program for the competency development of new nurses in a hospital emergency department. This program can be utilized to identify areas for improvement in hospital nursing care and serve as a leadership and management tool for nursing teams. Furthermore, it provides valuable insights for assessing nurse competencies and designing healthcare processes that align with the needs, circumstances, and environment of new nurses.

The design of this procedure is in line with Resolution No. 11/2019 of the General Council of Official Nursing Colleges of Spain [42], which regulates certain aspects of the professional practice of nurses in the field of urgencies and emergencies and in which in its framework of teaching action they describe that the following must be done:

- Detect both individual and group learning needs, taking into account the cognitive, psychomotor, and affective abilities and characteristics of individuals.
- Include in education programs specific aspects related to the prevention of complications and detection of signs and symptoms, as well as the essential initial action.
- Develop teaching activities aimed at nurses and other professionals aimed at strengthening competencies in care aspects in the field of urgencies and emergencies.

The existing literature does not reveal a program in our country similar to the one we present, which promotes the development of professional competencies directly within hospital units. Moreover, the evaluation of health professionals is not a common practice in public systems. In this context, our program addresses these gaps by providing tools for both self-evaluation and objective assessment by an external evaluator of nurses in hospital emergency services. Publications on mentoring programs for nursing developed in hospital emergency departments are scarce. Gayrama-Borines and Coffman describe a mentorship program in an emergency department that shares similarities with our onboarding program [43]. However, unlike ours, their program does not include an objective assessment of competencies. Several authors provide a solid context on the importance and effectiveness of transition and evaluation programs in the development of professional competencies in nursing, thus supporting the relevance and necessity of the program presented in our work [44,45,46].

Each tutoring program must be adapted to each hospital service as each of these has a specific profile and characteristics. Each unit is defined by specific processes, protocols, and procedures and by the use of specific computer applications. However, the transparency in this methodology allows the steps for its reproduction and applicability to be followed and reflects the tools for self-evaluation and objective evaluation of the program.

As for the duration of the tutoring program, it would be advisable to carry it out within a period of three months from the effective incorporation of the new recruit into the job. However, this period of time is flexible and can be adapted to the job where it is carried out and the current recruitment system of hospitals.

In our specific case, including a self-assessment instrument, although it could initially be considered a weakness, has become a strength of the program since the nurse perceives their improvement and is able to identify their areas of progress. Becoming aware of the competencies they must develop, as well as what their current level is, favors them to take responsibility for the process. According to what has been reflected in other studies, in order to make the person aware of their situation, continuous processes of constant reflection are required [47], and they consider self-evaluation as a good strategy to become aware of the level of competence [48] and a powerful technique to improve performance (Ross and Bruce [49]).

After a pilot study, we achieved very positive results in the self-evaluations of the nurses who were part of the program. The changes in the levels of nurses’ skills and competencies after tutoring showed that in a short period of time the novice nurse felt more qualified to work in the emergency department [50]. Our results reflect the benefits of a mentoring program on the competency development of novice nurses, as described by authors at the international level [51,52,53,54], and the high response rate of the questionnaires could indicate the commitment of nurses to the program [50].

The literature reflects that all mentoring programs improve nurse practice and retention, leading to improvements for patients and/or organizations [55]. An organized formal program, which includes comprehensive education and follow-up support, enhances the professionalization of frontline nurses and helps sustain a positive, constructive workplace environment [56].

A review published in 2020 notes that very few programs (8%, n = 6) explicitly identified the theoretical basis of their approaches [57]. Therefore, the purpose of this paper was to present a competency development program for nurses, reflecting the entire process. Our results offer tools to replicate the program in other units. The implementation of this program in additional units and hospitals will generate a very positive impact on their quality of care, being also more attractive to new professionals, by showing them support, promoting their competence development, and making them feel part of the team.

## 5. Strengths and Limitations

A strength of our program was the participation of experts during the in-depth analysis of the definition of the competency profile, as well as in the adaptation, understanding, and meaning of the evaluation items, which endorses the pertinence, relevance, usefulness, and clarity of the content of this program.

One limitation of the study is that it was administered in only one hospital. Additionally, the hiring system in our country, which sometimes involves short-term contracts, can hinder follow-up after training in some cases. Another limitation is the immediacy of hiring nurses, which allows little time for the program before they join the emergency service and requires immediate adaptation to the position. While our emergency unit has a dedicated person for tutoring, this may be a challenge in other hospitals, especially in the Spanish public health system, where such roles may not be available.

## 6. Conclusions

A tutoring program was developed to address a key need in healthcare: the onboarding of novice nurses in hospital emergency departments. The program primarily focused on the assessment and development of competencies. In the future, this program will not only serve as a valuable tool for the integration and support of new nurses but also has the potential to significantly impact quality improvement initiatives and patient safety. Its emphasis on person-centered values, including personalized integration and monitoring of nurses, development of necessary competencies for the role, and objective evaluation of professionals, positions it as a valuable asset for healthcare organizations seeking to retain staff and enhance overall care quality.

## Figures and Tables

**Table 1:** Rubric for assessing the level of competence in the knowledge and management of invasive mechanical ventilation in emergency situations.

	Level I: Beginner	Level II: Advanced Beginner	Level III: Proficient	Level IV: Efficient	Level V: Expert
Orotracheal intubation in emergency situations	Does not list all the necessary elements of a basic intubation. Does not recognize all the elements presented for difficult intubation. Does not correctly classify all necessary drugs. Does not know the correct intubation sequence (premedication → sedation → relaxation).	Meets two of these criteria: Lists all the necessary elements of a basic intubation. Recognizes some elements presented for difficult intubation. Correctly classifies six or more of the drugs needed. Does not know the correct intubation sequence (premedication → sedation → relaxation).	Lists all the necessary elements of a basic intubation (OTI). Recognizes all of the elements presented for difficult intubation. Correctly classifies six or more of the drugs needed. Does not know the correct intubation sequence (premedication → sedation → relaxation).	Lists all the necessary elements of a basic intubation. Recognizes all of the elements presented for difficult intubation. Correctly classifies all necessary drugs. Knows the correct intubation sequence. Has collaborated in real situations but not leading the nursing role.	Lists the necessary elements of a basic intubation and even takes into account those necessary in case of complication. Recognizes all of the elements presented for difficult intubation. Correctly classifies all necessary drugs. Knows the correct intubation sequence. Has experience in real-world situations as a nurse leader in these situations. Is able to train others.

**Table 2:** Rubric for evaluation of the level of competence in the knowledge and management of non-invasive mechanical ventilation (NIMV) and Aerogen^®^ vibrating mesh nebulization system.

	Level I: Beginner	Level II: Advanced Beginner	Level III: Proficient	Level IV: Efficient	Level V: Expert
Non-invasive mechanical ventilation + Aerogen^®^ nebulization	Does not know the Aerogen^®^ device or how it is assembled. Does not know the material needed to assemble a NIMV circuit or how to assemble it. Does not know how to interpret indicators from the ventilator monitor or solve the incidents shown.	Knows the Aerogen^®^ device and the material required for its use but not its assembly. Knows the material needed to assemble a VMNI circuit but does not know how to assemble it or has any doubts during assembly. Does not know how to interpret indicators from the ventilator monitor or solve the incidents shown.	Knows the Aerogen^®^ device. Capable of assembling it without instructions properly. Knows the material needed to assemble a VMNI circuit. Assembles it without hesitation. Does not know how to interpret indicators from the ventilator monitor or solve the incidents shown.	Knows the Aerogen^®^ device. Capable of assembling it without instructions properly. Knows the material needed to assemble an NIMV circuit. Carries out the assembly properly autonomously. Knows how to interpret indicators from the ventilator monitor and solve incidents shown.	Knows the Aerogen^®^ device. Capable of assembling it without instructions properly. Knows the material needed to assemble an NIMV circuit. Assembles properly autonomously. Interprets the indicators present in the ventilator monitor. Troubleshoots and interprets simple issues. Has experience in real situations. Is able to train others.

## Data Availability

The dataset is available on request from the authors.

## Supplementary Materials

The following supporting information can be downloaded at https://www.mdpi.com/article/10.3390/nursrep14030176/s1: File S1.A Table. Definition of all the competencies selected for the tutor’s competency profile, as well as the level required for each of them and the three objectifiable behaviors associated with each level; File S1.B Reception and tutoring program aimed at new nurses in hospital emergency department. Welcome program; File S1.C Individual program monitoring form; File S1.D Self-assessment questionnaire on emergency nursing skills and competencies Tool.

## Public Involvement Statement

No public involvement in any aspect of this research.
