# Supplementary material for: Design, Implementation, and Adaptation of a Tutoring Program for the Competency Development of New Nurses in a Hospital Emergency Department
Source: Nurs Rep. 2024 Sep 14;14(3):2370–84. doi: 10.3390/nursrep14030176 (PMC11417872; doi:10.3390/nursrep14030176)
Supplement: Supplementary file 1 [file nursrep-14-00176-s001.zip › nursrep-3137617-supplementary.pdf]

SUPPLEMENTARY MATERIAL 1.A: DEFINITION OF ALL THE COMPETENCIES SELECTED FOR THE TUTOR'S COMPETENCY PROFILE, AS WELL AS THE LEVEL REQUIRED FOR EACH OF THEM AND THE THREE OBJECTIFIABLE BEHAVIORS ASSOCIATED WITH EACH LEVEL.

COMPETENCE	DEFINITION OF COMPETENCE	LEVEL DEFINITION	ASSOCIATED BEHAVIORS
GENERIC COMPETENCE<br><br>Commitment (Level 4)	Ability to feel the hospital's objectives as one's own and to fulfill personal, professional and organizational obligations. Ability to support and implement decisions aimed at achieving common objectives. Prevent and overcome obstacles that interfere with the achievement of organizational goals. It implies adherence to the values of the organization.	Ability to comply with the organization's strategic plan and generate in all its members the ability to feel the objectives as their own. Ability to demonstrate respect for organizational values and culture and people and to motivate others to do the same. It also implies the ability to exceed the expected results in its management. He is also a role model for his team for his discipline and efficiency.	Verify the achievement of the objectives in the team, motivating them and making them participate to generate commitment and identification.<br><br>It designs and provides tools for the execution, monitoring and control of planned actions, in order to control the progress of its processes to achieve the proposed objectives, in the constant search for quality and efficiency improvement.<br><br>It is a guide for action and decision-making in every situation. Make your team feel part of every accomplishment.
GENERIC COMPETENCE<br><br>Quality & Innovation (Level 3)	Ability to optimize available resources: people, materials and provide innovative ideas, approaches or solutions in relation to the assigned intervention. This innovative contribution will make it possible to improve, modernise or optimise the use of available resources.	Ability to propose improvement/innovation plans that allow you to establish yourself as a benchmark for your team in terms of quality and innovation.	It designs and implements monitoring and control mechanisms, aimed at measuring quality.<br><br>Promotes the continuous improvement of processes and services in its work team.<br><br>They have extensive knowledge of the organization and their area of expertise and generate mechanisms for exchanging and taking advantage of the knowledge and experience of each team member.

COMPETENCE	DEFINITION OF COMPETENCE	LEVEL DEFINITION	ASSOCIATED BEHAVIORS
GENERIC COMPETENCE<br><br>Ethics<br>(Level 3)	Ability to feel and act in accordance with moral principles, codes of ethics, and socially accepted behaviors. Carrying out professional internships in accordance with the mission, vision and values of the hospital.	Ability to direct the area under his/her charge and act on the basis of moral values, good customs and organizational practices and establish a framework for oneself and for the entire area based on respect for both the organization's policies and moral values and principles. Ability to prioritise values and good manners over personal interests and to establish labour, commercial or inter-area relationships based on principles and respect.	It conveys the vision and mission of the hospital based on moral values.<br><br>It establishes a framework that respects both the hospital's policies and moral values, good customs and professional practices.<br><br>He is a role model for his team for his ethical behavior.
GENERIC COMPETENCE<br><br>Orientation to the user and professionals<br>(Level 3)	Ability to act with sensitivity to the needs of users and professionals, current or potential. It implies a permanent vocation for service, adequately understanding their demands and generating effective solutions to their real and potential needs.	Ability to anticipate the needs and expectations of users and professionals and constantly look for ways to solve them. Ability to propose improvement actions in their area, aimed at increasing the level of satisfaction of users and professionals and providing excellent solutions to their needs. Ability to establish long-lasting relationships with users and professionals based on trust.	It adapts products and services to the current and potential needs of users and/or professionals.<br><br>He is personally responsible for correcting the problems and needs of users and/or professionals.<br><br>It promotes the flow of information and ideas at multiple levels through formal and informal practices. Publicize successes.

COMPETENCE	DEFINITION OF COMPETENCE	DEFINICIÓN DEL NIVEL	ASSOCIATED BEHAVIORS
SPECIFIC COMPETENCE<br><br>ANALYTICAL THINKING (LEVEL 3)	Ability to understand a situation or changes in the environment, identify its parts and organize them systematically, in order to determine their interrelationships and establish priorities for action.	Ability to understand complex situations or problems and break them down into their various components. Ability to interrelate these components, establish complex causal links, and recognize the possible causes of an event, or the consequences of an action or chain of events. Ability to identify the relationships between the different elements of a problem or situation in order to anticipate obstacles and plan the next steps. Ability to develop alternative courses of action in line with the possible derivations of the situation.	It identifies chains of events, the facts, causes and consequences that lead to a problem, and can generate successful solutions to solve them.<br><br>When faced with a problem, it recognises pre-existing situations and proposes different alternatives, taking into account the consequences and impact of each of them in order to develop preventive proposals.<br><br>It produces accurate and easy-to-understand reports, interpreting and simplifying the complexity of the information it handles so that it can be used by the various areas of the organization.
SPECIFIC COMPETENCE<br><br>TEAMWORK (Level 3)	Ability to collaborate with others, be part of a group and work with other areas of the organization with the purpose of achieving, together, the organizational strategy, subordinating personal interests to group objectives. It involves having positive expectations of others, understanding others, and generate and maintain a good working environment**.**	Ability to foster a spirit of collaboration in their area and promote the exchange of other sectors of the organization. It involves expressing satisfaction with the successes of others, whether or not they belong to the immediate work group. Subordinate personal interests to group objectives, in order to achieve organizational goals. Ability to establish themselves within their area as an example of collaboration and cooperation, understand others and maintain a good working environment.	Devise and implement alternative modalities of teamwork in order to add value to group outcomes.<br><br>Encourage and motivate others. Resolve your team's conflicts.<br><br>It acts to generate a friendly working environment, good climate and cooperation.

COMPETENCE	DEFINITION OF COMPETENCE	LEVEL DEFINITION	ASSOCIATED BEHAVIORS
SPECIFIC COMPETENCE<br><br>COMMUNICATION EFFECTIVE (Level 3)	Ability to actively listen and clearly transmit to users, family members and professionals, the appropriate information to their needs and demands and to maintain open communication channels that guarantee their rights and duties dictated by the organization.	Ability to listen, understand and clearly transmit to users, family members and professionals, the appropriate information to their needs and demands. Ability to adapt their communication style to language and cultural barriers. Ability to develop networks that are useful in creating a positive environment of intercommunication.	He communicates his ideas in a clear, efficient and fluid way, getting his audience to understand his message and impacting them in the sense they want.<br><br>Adjust the language to the terminology, characteristics and needs of your interlocutor or the group with which you are interacting.<br><br>Listen to others with empathy, taking care to understand their points of view and avoiding preconceptions and judgments.
SPECIFIC COMPETENCE<br><br>LEARNING CONTINUOUS (Level 3)	Ability to search, acquire, and share useful information for problem solving, utilizing the full potential of the hospital and adding value to it. It includes the process of acquiring new skills.	Ability to act as a benchmark in their area and in the hospital. He offers his experience and knowledge to solve problems in other areas as well as presenting innovative and creative solutions to diverse situations.	It undertakes actions to disseminate new methodologies and technologies in the hospital.<br><br>He is a benchmark in the hospital in his specialty. He shares knowledge with his team and fosters a spirit of innovation, updating and internalization of knowledge.<br><br>They expand their knowledge beyond their immediate area of work and cooperate with other areas of the hospital when their knowledge is required for problem solving.

COMPETENCE	DEFINITION OF COMPETENCE	LEVEL DEFINITION	ASSOCIATED BEHAVIORS
TECHNICAL COMPETENCE<br><br>TRAINER<br>(LEVEL 4)	Ability to train others in both knowledge and skills. It implies a genuine effort to foster the long-term learning and/or development of others, beyond their specific, day-to-day responsibility. The development to be achieved in others will be on the basis of individual effort and according to the position that the other person currently occupies or is expected to occupy in the future.	Ability to offer honest, respectful and objective feedback to their collaborators, pointing out their strengths and weaknesses along with the most effective development needs; Promote independence, develop skills (knowledge and skills) and strengthen them in order to foster long-term learning and growth. It involves monitoring each of your employees individually, providing them with effective advice, and considering all variables related to (both the organization and the employee).	Assign your employees tasks that are challenging, encouraging them to develop new skills.<br><br>He is a reference for his collaborators and valued for his predisposition and clarity when it comes to setting objectives for them, both in tasks and in their possibilities for growth.<br><br>You easily detect strengths and weaknesses of your employees, creating the opportunities for training and development required based on those strengths and weaknesses.
COMPETENCIA TÉCNICA<br><br>COLABORACIÓN<br>(NIVEL 2)	Ability to provide support to others (peers, superiors and collaborators), respond to their needs and requirements, and solve their problems or doubts, even if they have not been expressly expressed. It involves acting as a facilitator for the achievement of goals, in order to create relationships based on trust.	Ability to actively support and collaborate with members of their own area through a clear predisposition to help others, even before they have expressly expressed the need for collaboration. Ability to listen to the requirements of others and to help them meet their goals, without neglecting their own.	Listen to the requirements of others.<br><br>Shows interest in collaborating with others to achieve organizational goals.<br><br>It sustains good interpersonal relationships and builds trust.

COMPETENCE	DEFINITION OF COMPETENCE	LEVEL DEFINITION	ASSOCIATED BEHAVIORS
COMPETENCIA TÉCNICA<br><br>EMPOWERMENT (NIVEL 3)	Ability to empower the work team and share both the successes and the negative consequences of the results with all collaborators. Ability to undertake effective actions aimed at improving and enhancing people's talent, both in terms of knowledge and skills. Ability to obtain the best results, achieve group integration and take advantage of the diversity of team members to achieve added value beyond the service provided. It involves setting clear, measurable performance goals and assigning responsibilities accordingly.	Ability to design and implement working methods that allow the different teams to be empowered. Ability to take effective actions to improve and enhance the talent of people in their area, both in terms of knowledge and skills. Ability to achieve and exceed the objectives set for their area and take advantage of the diversity of all employees in order to achieve added value beyond the organizational strategy. It involves implementing clear and measurable processes and performance objectives, and assigning the corresponding responsibilities to the different collaborators in their charge.	Assign responsibilities to each of your team members, taking into account their skills<br><br>He has a good knowledge of the capabilities of his collaborators and is concerned about their development for future responsibilities.<br><br>It gives feedback on performance to each member, stimulating and motivating their continuous development.
TECHNICAL COMPETENCE<br><br>TEMPER AND DYNAMISM (LEVEL 3)	Ability to act with serenity, determination, firmness, enthusiasm and perseverance in order to achieve challenging objectives or to carry out actions and/or undertakings that require commitment and dedication. It involves maintaining a high level of performance in all situations and with diverse interlocutors.	Ability to act with determination, firmness, perseverance and commitment in order to carry out the actions that allow them to achieve the objectives assigned to the area they lead, overcome demanding situations successfully and maintain at all times a level of performance in accordance with the standards established for their position. It implies constituting oneself This is an example for your area in terms of firmness and dynamism.	Maintains control of the area in adverse circumstances.<br><br>He is firm and constant in achieving his work objectives.<br><br>Maintains an optimal level of performance.

SUPPLEMENTARY MATERIAL 1.B: RECEPTION AND TUTORING PROGRAM AIMED AT NEW NURSES IN HOSPITAL EMERGENCY DEPARTMENT. PHASE 1: WELCOME PROGRAM.

Structure:

1.1 Reception & Welcome

From the moment that the hiring is effective and the unit has proof of the name of the new member of the team, the supervisor of the unit contacts the tutor, indicates the details of the new nurse and, at this moment, the plan is activated.

1.2 Phone call

In this first call, the contact between the tutor and the newly hired nurse takes place. There is a presentation of the figure of the tutor and a brief explanation of the tutoring process that is offered.

An interview is conducted to find out the starting point (previous experience as a nurse, experience in emergency services) and a date is set for a first in-person welcome meeting.

Sending documentation: After the initial call, a welcome email is sent to the email provided. It includes relevant information on procedures to be carried out before incorporation (request for access codes and administrative procedures) as well as informative and consultation documents, with practical information to know how the service works:

Welcome guide: information on the administrative functioning of the unit, management of human resources and organization of staff and service.

Hospital Clinical Post (PCH) Guide, a computer program common to all public EDs in our region.

Manual of how to operate in observation rooms, since these are the first locations where the new nurse is going to work as it is an area more similar to hospitalization.

Practical pocket guide that collects the information in an accessible and practical way for the recording of activities in the computer system and the most common tasks to place yourself in the first days of work.

1.3 Welcome Session:

This session is tailored to the profile of the novice nurse, as we analyse the nurse's profile in a personalised way to find out their learning needs, the elements that are not unknown to them and those that we must have an impact on.

In the first face-to-face session, new staff is instructed on the use of the computer manager of our PCH unit, with which the registration of all care activity is developed and is the key element for functional development in the unit.

In that same session, training is carried out on important elements of the unit, including the basic equipment, the automated drug dispensing system, the infusion pumps, the electrocardiogram with transmission to the electronic medical record and the venous and arterial gas analyzer. In our unit, an inventory of the equipment has been carried out, and each device consists of a physical QR with a training video on its use and the most frequent doubts about its use, each video is hosted on a secure corporate page, created by the hospital's computer scientists.

The work dynamics of the different locations of our service are analysed with the novice nurse, since there is a great variability between the care work in an observation room and that carried out in the first aid boxes.

From this moment on, several channels of active communication with the tutor remain open, e-mail, face-to-face appointments, telephone appointments or mobile application messaging.

1.4 Nurse Library Availability

All staff have at their disposal, on all the computers of the unit as a network, a Nurse Library developed in the service. This Library is a compilation of documents and videos on procedures in force at the Hospital and that may be relevant to the development of care within the unit, as well as documents and guides to support clinical practice that can be consulted by all staff.

1.5 Field work accompaniment

It is planned that the incorporation into the different areas of work, within our unit, will be gradual. This facilitates the acquisition and fixation of the different care circuits and processes. In order to comply with the progressive adaptation of the new staff to the position, the supervisor must reorganize the structural staff, to guarantee the coverage with regular staff, of all the locations of our unit, and respect the criteria of accompaniment of the program and the adaptation period of the novice.

Initially, new nurses begin their first days in observation rooms, due to their similarity to hospitalized patient care and previous work experiences, and once adapted to this area, they begin their process in first care boxes.

In order to access the work carried out in the resuscitation area and triage area, it is essential to have specific, regulated and accredited training in triage. This training is provided by the centre to all staff who have been in the service for one year and are expected to be continuous. It is complemented by a specific training offer in the resuscitation area area given by the service's own staff.

The tutor is in charge of providing accompaniment according to the needs of each worker during the first days in the different locations, especially in the observation room and general boxes. The aim of this gradual accompaniment is to facilitate the process of adaptation to the different work areas and that the worker's self-confidence is acquired from a base of safety and confidence, minimising as much as possible the anxiety and stress that it may entail. In addition, this promotes the maintenance of the line of work marked in each area in terms of processes, quality of care and patient safety.

1.6 Control of the adaptation process

The entire process of reception and accompaniment is recorded in individual files and an excel table with the professional's data (supplementary material File 1. C Individual program monitoring form). It includes a training program with scheduled and follow-up sessions upon arrival at the unit, after the reception meeting and 15, 30, 45 and 60 days after joining. The questionnaire on technical competencies and skills sent and collected to analyse the specific situation of the staff at the time of their incorporation are carried out again at 30 days and 60 days, thus obtaining an evolutionary curve that can show us the level of adaptation and the perceived competency learning curve.

In addition, the results of the questionnaires after this period of time are reviewed in person with the interested party in a new interview with the tutor to comment on the real evolution observed and establish new objectives, jointly, that help to improve the most deficient competencies and skills.

SUPPLEMENTARY MATERIAL 1.C: INDIVIDUAL PROGRAM MONITORING FORM.

INDIVIDUAL REGISTRATION SHEET

NAME			
MAIL		TELEPHONE	
CONTRACT TYPE			
CONTRACT START DATE		END DATE OF CONTRACT	
PIXYS PASSWORD*		YEAR DEGREE	
PCH PASSWORD**			

*Pixys (Automated Drug Dispensing System) **PCH (Hospital Clinical Post)

EXPERIENCE IN THIS ED***	
EXPIENCIA EN OTRO ED	
EXPERIENCE IN THIS HOSPITAL	
TRIAGE CERTIFICATE	

*** ED (Hospital Emergency Department)

	COMPLETION DATE
WELCOME MEETING	
TELEPHONE NETWORK GROUP	
INITIAL QUESTIONNAIRE	
30-DAY QUESTIONNAIRE	
60 DAY QUESTIONNAIRE	
30-DAY MEETING	
60-DAY MEETING	

FIRST SHIFTS	DATE / SHIFT	LOCATION	HOSTING

BEFORE THE WELCOME MEETING		AFTER WELCOME MEETING		AT 15 DAYS		AT 30 DAYS		AT 45 DAYS		AT 60 DAYS	
Welcome guide		Initial Questionnaire		Monitoring protocol		30-day questionnaire		Advanced Triage		60 Day Questionnaire	
Pocket guide		Equipment videos		Cardiopulmonary resuscitation trolley		Restraint Poster		ALS Document*		PU Document**	
PCH Manual		Transfusion Protocol		First Day Assistance Box		Reservoir handling		IV lines protocol		CVP Training	
Rooms Manual		FAQ								NIMV Training****	
Telephone Network Group		Library Index									

*ALS (Advanced Life Support) **PU (Pressure Ulcer) ***CVP (Central Venous Pressure) ****NIMV (Non-Invasive Mechanical Ventilation) *****TC PM (Transcutaneous Pacemaker)

WORKSHOPS	
ALS Case	
Subcutaneous reservoir	
Orotracheal intubation (OTI)	
NIMV	
Defibrillator/TC PM*****	
STROKE Session	
Mechanical Restraint	

PROPOSED OBJECTIVES (Start/End Date)

SUPPLEMENTARY MATERIAL 1.D: SELF-ASSESSMENT QUESTIONNAIRE ON EMERGENCY NURSING SKILLS AND COMPETENCIES.

AGE: GENDER: PROFESSIONAL EXPERIENCE (in years and months):

IS THIS YOUR FIRST HOSPITAL'S EMERGENCY CONTRACT? YES / NO

HOW LONG HAVE YOU BEEN EMPLOYED IN THE EMERGENCY ROOM (in months) _________

**Technical Skills**	**Expert**	**Efficient**	**Competent**	**Advanced Beginner**	**Beginner**
1. Triage					
2. Re-triage					
3. Transfer patients					
4. Advanced Life Support					
5. NIMV/IMV handling (a)					
6. Suture					
7. Immobilization cast/bandage					
8. Peripheral IV line, Central Peripheral Access and intraosseous route					
9. Burn Cure					
10. Stomach pumping					
11. Psychiatric Patient Restraint Management					
12. Minor surgery assistance					
13. Knowledge and handling of the unit's equipment					
14. Knowledge and handling of drugs in the unit					
15. Management of polytrauma patients					
16. STROKE CODE Management					
17. INFARCTION CODE HANDLING					
18. SEPSIS CODE Management					
19. Protocol management of severe-massive bleeding					
20. Advanced Triage Management of Acute Urine Retention					
21. Advanced triage management of voiding syndrome					
**COMPETENCES**					
1. INITIATIVE/AUTONOMY					
2. TEAMWORK					
3. COMUNICACIÓN EFICAZ					
4. PRESSURE TOLERANCE					

a: Non-invasive mechanical ventilation/invasive mechanical ventilation.
